# A history of study and new records of terrestrial enchytraeids (Annelida, Clitellata, Enchytraeidae) from the Russian Far East

**DOI:** 10.3897/zookeys.955.53106

**Published:** 2020-08-05

**Authors:** Maksim I. Degtyarev, Iurii M. Lebedev, Ksenia G. Kuznetsova, Konstantin B. Gongalsky

**Affiliations:** 1 Severtsov Institute of Ecology and Evolution, Russian Academy of Sciences, 33 Leninskij prosp., Moscow, 119071, Russia Severtsov Institute of Ecology and Evolution, Russian Academy of Sciences Moscow Russia; 2 Lomonosov Moscow State University, GSP-1, Leninskie Gory, Moscow, 119992, Russia Lomonosov Moscow State University Moscow Russia; 3 Skolkovo Institute of Science and Technology, 30–1 Bolshoy Boulevard, Moscow, 121205, Russia Skolkovo Institute of Science and Technology Moscow Russia; 4 Federal Research and Clinical Center of Physical-Chemical Medicine, Federal Medical Biological Agency, 1a Malaya Pirogovskaya, Moscow, 119435, Russia Federal Medical Biological Agency Moscow Russia

**Keywords:** biodiversity, COI, potworms, soil fauna, 12S rRNA, 16S rRNA

## Abstract

A list of terrestrial enchytraeids of the Russian Far East is compiled based on literature and extensive field data collected by the authors in 2019. A database has been created consisting of geographic coordinates, habitat type, species, and data source. For some species collected by the authors, barcoding using COI, 16s, and 12s rRNA genes has been performed. In total, there are at least 62 species of enchytraeids belonging to 12 genera. Seven species (*Achaeta macroampullacea*, *Cognettia sphagnetorum*, *Enchytraeus dichaetus*, *Fridericia cusanica*, *Globulidrilus riparius*, *Marionina southerni*, *Mesenchytraeus gigachaetus*) are reported in the Russian Far East for the first time. *Cognettia sphagnetorum* and *F. cusanica* are most probably introduced. Taxonomic and biogeographical remarks on some of the species found and differences from the original descriptions are provided. Some of the specimens may be undescribed species, but this requires a more in-depth examination. The Russian Far East, especially its southeastern part, is of great interest as a possible location for new species of enchytraeids.

## Introduction

The Russian Far East (hereafter referred to as RFE) ranges in climate, from arctic deserts in the north to dry steppes and broadleaf forests in the south. In this paper, RFE is equated with the Far Eastern Federal District of Russia, which includes the Republic of Buryatia, Sakha Republic (Yakutia), Kamchatka, Khabarovsky, Primorsky, and Zabaykalsky Krais, Amur, Magadan and Sakhalin Oblasts, Chukotka Autonomous Okrug, and Jewish Autonomous Oblast. This region occupies 40.6% of the territory of Russia (Rosstat 2019). On the Pacific coast, the climate is monsoon, which is most pronounced in the south and weakens to the northeast. Summer here is moderately warm and rainy, and winter, although not very snowy, is cold, due to the spread of cold air from the interior of Siberia. More inland, the climate becomes more continental, with warm summers and very cold winters. The northern regions of the RFE, adjacent to the Arctic Ocean, are characterized by cold summers and cold winters. Landscapes also vary from mountain ranges in the northeast to the plains along the Amur River in the south. Part of the territory is represented by islands and archipelagos, such as Sakhalin, the Kuril Islands, the New Siberian Islands, and Wrangel Island (Krestov 2003). The large area of the RFE contributes to the diversity of biomes. Arctic desert and tundra are replaced to the south by a wide strip of coniferous forests and then by forest-steppes and steppes. In the southeast of the RFE, in Primorsky Krai, broadleaf and mixed forests are represented. Diversity of biomes leads to great diversity of flora and fauna, especially that of invertebrates (Storozhenko et al. 2002; Ryabinin 2015). However, general knowledge about the soil biodiversity of this area is rather poor and fragmentary.

The Enchytraeidae are a family of terrestrial oligochaetes consisting mainly of small unpigmented annelid worms. Enchytraeids inhabit all continents and are perhaps the most widespread representatives of the Clitellata (Erséus 2005). An ecologically plastic group, enchytraeids are found in a wide range of habitats. Various species of the family are broadly represented in soil and in the splash zone on seacoasts; they are also found in freshwater and marine sediments (Erséus and Rota 2003; Erséus 2005). Twenty of the 35 valid genera of enchytraeids are found in soil (Coleman et al. 2018; Timm and Erséus 2020), where they are important in the decomposition of organic matter (Römbke et al. 2005).

At the same time, the RFE, as well as the territory of Russia as a whole, the enchytraeid fauna has been poorly studied, and this situation has persisted since the 1980s (Nurminen 1982). Thus, researching enchytraeid diversity of the RFE has both inventory and biogeographic significance.

In the last decade, many studies have been conducted on the fauna of enchytraeids of the territories geographically close to the RFE: Manchuria (Chen and Xie 2015), South Korea (Dózsa-Farkas and Hong 2010; Dózsa-Farkas et al. 2015), and Japan (Torii 2012). In contrast, there is a knowledge gap for enchytraeids in the RFE itself, some studies have been conducted. Some species were described from materials collected by Russian polar expeditions at the beginning of the 20^th^ century (Michaelsen 1901, 1916; Cejka 1910, 1912, 1914). After many years, Piper et al. (1982) reported the enchytraeid fauna at several points on the coast of northeastern Russia. Nurminen (1980) found several new species of enchytraeids in samples sent from Primorsky Krai. Ganin (1984) suggested the possibility of finding even more new species in the RFE. In 1994, a Swedish-Russian expedition took samples, including for enchytraeid extraction, in the Palaearctic tundra. Some of their sampling sites were located in the RFE (Christensen and Dózsa-Farkas 1999).

In the summer of 2019, we have collected much material from across the RFE, between the city of Magadan (northern coast of the Okhotsk Sea) and the southernmost areas of Primorsky Krai. The identity of some species was confirmed with the help of molecular analysis of the genes COI, 12S rRNA, and 16S rRNA.

In this study we summarize currently available data on the fauna of terrestrial enchytraeids of the RFE, both from the literature and our own new data.

## Materials and methods

To estimate terrestrial enchytraeid diversity of the RFE, a database has been created. It includes the following attributes: geographic coordinates of sampling sites, habitat type, species, and data source. Data on enchytraeids from marine and freshwater environments were not included.

**Data from literature.** We included data collected by other researchers. These data are mostly from tundra and taiga habitats (see below). Although there are numerous reports of enchytraeids in soil samples in the soil-zoological literature, we only included those studies where enchytraeid species were identified.

**Figure 1.**
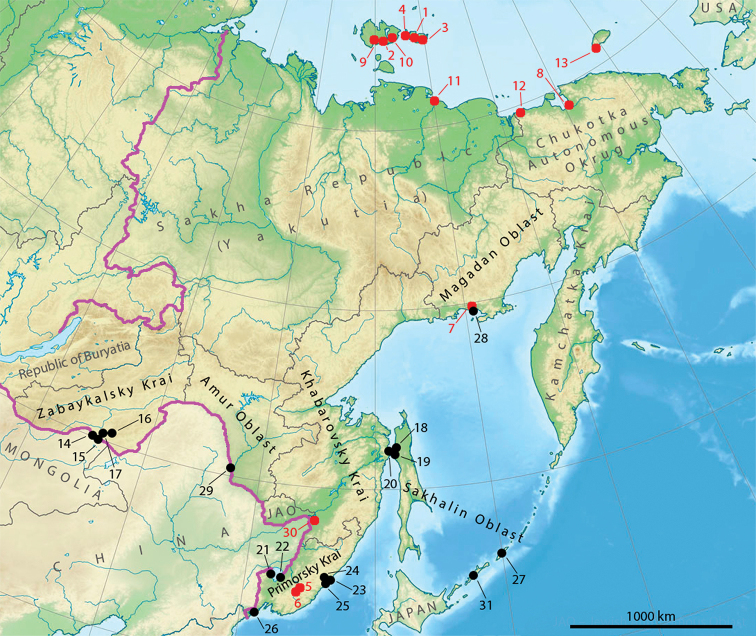
Map of enchytraeid record locations in the Russian Far East. Black dots represent own data sampled in July-August 2019. Red dots are data from the literature. Purple line depicts the administrative border of the Far Eastern Federal District. JAO – Jewish Autonomous Oblast.

**Our data.** We collected our material during July and August 2019 in Khabarovsky, Primorsky, and Zabaykalsky krais, as well as Amur, Magadan, and Sakhalin oblasts (Fig. 1). The investigations were carried out in steppes, broadleaf and coniferous forests, and azonal habitats like floodplains and meadows. Within each locality (see Fig. 1), five samples each 5 cm in diameter were collected and brought to the laboratory in Moscow. They were stored at 4 °C until the extraction. Enchytraeids were extracted from soil using the wet funnel method (Römbke 1995), identified *in vivo*, and then preserved in 96% alcohol.

Species were identified according to Schmelz and Collado (2010) or, for species not found in Europe, by comparison with the original descriptions. Taxonomy follows Schmelz and Collado (2012, 2015), excluding genera and species described after 2015.

Some samples were selected for genetic analysis. The DNA extraction was performed using “ExtractDNA blood” kits (Evrogen, Russia). In each sample the entire enchytraeid body was used for DNA extraction. We amplified one or two of three mitochondrial regions: 12S rRNA, 16S rRNA, and cytochrome oxidase I (COI) genes. The COI fragment is the standard DNA barcode for animals (Hebert et al. 2003). The PCR mixture contained 1–3 ng of the DNA matrix, 0.1 μM of each primer, and the precast PCR mixture from Evrogen (Russia) according to the manufacturer's instructions. The primers were synthesized by Evrogen (Russia) as well. For the COI gene the primers were LCO1490 (Folmer et al. 1994) (5'-GGTCAACAAATCATAAAGATATTGG-3') and COI-E (Bely and Wray 2004) (5'-TATACTTCTGGGTGTCCGAAGAATCA-3') and the amplification program was: 95 °C for 5 min, 35 cycles of 95 °C for 40 sec, 45 °C for 45 sec, 72 °C for 60 sec, and finally, 72 °C for 8 min, resulting in a fragment of about 608 bp. For the 12S rRNA gene the primers were 12S-E1 (5'-AAAACATGGATTAGATACCCRYCTAT-3') and 12SH (5'-ACCTACTTTGTTACGACTTATCT-3') (Jamieson et al. 2002), and the amplification program was: 94 °C for 2 min; 35 cycles of 94 °C for 15 sec, 50 °C for 15 sec, 72 °C for 90 sec, and finally 72 °C for 5 min, resulting in a fragment of about 322 bp. For the 16S rRNA gene the primers were 16SAR-L (5'-CGCCTGTTTATCAAAAACAT-3') and 16SBRH (5'-CCGGTCTGAACTCAGATCACGT-3') (Palumbi et al. 2002), and the amplification program was: 95 °C for 5 min, 35 cycles each of 95 °C for 30 sec, 50 °C for 30 sec, 72 °C for 60 sec, and finally, 72 °C for 8 min resulting in a fragment of about 423 bp.

The phylogenetic analysis for species of *Fridericia* Michaelsen, 1889 was conducted using the maximum likelihood (ML) method. *Enchytraeus albidus* Henle, 1837 was chosen as an outgroup. Phylogenetic analysis involved 25 16S rRNA nucleotide sequences; 20 of them were obtained from NCBI GenBank. The sequences were aligned using Clustal Omega (Sievers et al. 2011). There was a total of 409 positions in the final alignment. GTR model of nucleotide substitution with Gamma distribution and invariable sites was chosen using the BIC criterion (Nei and Kumar 2000). The initial tree was obtained automatically by applying NJ and BioNJ algorithms and then the topology with superior log likelihood value was selected. All positions containing gaps and missing data were eliminated. Bootstrap replications value was set to 10000. The ML analysis was performed in MEGA X (Kumar et al. 2018).

The phylogenetic analysis for species of *Bryodrilus* Ude, 1892 was conducted using the Bayesian inference (BI) method. *Henlea perpusilla* Friend, 1911 was chosen as an outgroup. Phylogenetic analysis involved 10 COI mDNA nucleotide sequences; nine of them were obtained from NCBI GenBank. The sequences were aligned using Clustal Omega (Sievers et al. 2011). There was a total of 528 positions in the final alignment. GTR model of nucleotide substitution with Gamma distribution was chosen in TOPALi v. 2 (Milne et al. 2009) using the AIC criterion (Akaike 1974). The analysis was run with 4 chains for 20,000,000 generations, sampling each 1000 generations and burning in 25%. The BI analysis was performed using MrBayes v. 3.2 software package (Ronquist et al. 2012). A list of all DNA sequences originally obtained in this study is provided (Table 1).

**Table 1.** DNA sequences of enchytraeid species originally obtained in this study. Species, localities and GenBank accession numbers are given in columns.

Species	Locality	GenBank accession numbers		
		12S	16S	COI
*Bryodrilus* sp. A	6 km north-east of Terney, Primorsky Krai, Russia. *Quercus*-*Betula* forest. Cambisol soil. 45°02'29N, 136°40'56E.	MT241728	N/A	MT237714
*Fridericia bulboides* type 'a'	Near Serebryanka river, Primorsky Krai, Russia. Sparse *Betula* forest. Cambisol soil. 45°06'42N, 136°31'51E.	MT241729	MT232978	N/A
*Fridericia bulboides* type 'c'	same	MT241730	MT232979	N/A
*Fridericia bulboides* type 'c'	same	MT241731	MT232980	N/A
*Fridericia bulboides* type 'd'	same	N/A	MT232981	N/A
*Mesenchytraeus gigachaetus*	6 km north-east of Terney, Primorsky Krai, Russia. *Quercus*-*Betula* forest. Cambisol soil. 45°02'29N, 136°40'56E.	MT214330	N/A	MT160424

### List of localities with confirmed enchytraeid data

The habitats are described and the geographical coordinates (WGS 84) are provided with varying degree of precision depending on the information given in the original source. For the sites based on literature data, the respective references are given. The absence of a literature reference presumes that enchytraeids from this site were identified by us. In the absence of an accurate geographical reference (literature from the beginning of the 20^th^ century), a point on the map (Fig. 1) shows only approximate place of origin of the sample. In the commented list of terrestrial enchytraeids of the RFE, the localities are given for each species, in accordance with description of the sampling sites.

### Sites for which data were available from the literature:

[1] New Siberian Islands. Between 74°30'–76°N, 137–150°E (Cejka 1910).

[2] Kotelny Island. Between 74°30'–76°N, 137–144°E (Cejka 1912).

[3] New Siberian Islands. Between 74°30'–76°N, 137–150°E (Cejka 1914).

[4] New Siberian Islands. Between 74°30'–76°N, 137–150°E (Michaelsen 1901).

[5] Primorsky Krai, Upper Ussuri station ca 35 km NE of Chuguyevka. Litter of *Ulmus* wood in a mountain stream valley. Ca 44°23'N, 134°12'E (Nurminen 1980).

[6] Primorsky Krai, Upper Ussuri station. Litter of *Picea* and *Abies* forest with *Dryopteris* and *Pleuzorium*. Ca 44°04'N, 133°56'E (Nurminen 1980).

[7] Taiga forests near Magadan (with azonal habitats: stream bank and riparian birch forest). Ca 59°30'N, 151°50'E (Piper et al. 1982).

[8] Tundra at the Chaun Bay research station (including mesic heath, mountain area and wet meadow). Ca 68°44'N, 170°36'E (Piper et al. 1982).

[9] Kotelny Island. 75°03'30"N, 140°11'24"E (Christensen and Dózsa-Farkas 1999).

[10] Faddeyevsky Island. 75°29'36"N, 143°44'30"E (Christensen and Dózsa-Farkas 1999).

[11] Lopatka Peninsula. 72°11'06"N, 148°26'18"E (Christensen and Dózsa-Farkas 1999).

[12] NE Kolyma Delta. 69°21'18"N, 163°34'48"E (Christensen and Dózsa-Farkas 1999).

[13] Wrangel Island. 70°57'24"N, 179°33'24"E (Christensen and Dózsa-Farkas 1999).

[30] Khabarovsky Krai, oak forest, Khekhtsir mountain range, 20 km south of Khabarovsk. Ca 48°11'N, 134°51'E (Ganin 1984).

### Sites where material was collected by the authors:

[14] Near Ust-Imalka, Daurian Nature Reserve, Zabaykalsky Krai. Dry steppe with *Stipa* and *Leymus*. Kastanozem soil. 50°11'02"N, 115°26'09"E. 27.07.2019.

[15] South of Kulusutay, Daurian Nature Reserve, Zabaykalsky Krai. Dry steppe with *Stipa* and *Leymus*. Kastanozem soil. 50°08'36"N, 115°41'36"E. 28.07.2019.

[16] Borzya river bank, Zabaykalsky Krai. Periodically flooded meadow with *Carex*. Fluvisol soil. 50°31'23"N, 116°45'20"E. 25.07.2019.

[17] Adun-Chulun mountains, Daurian Nature Reserve, Zabaykalsky Krai. Mountain steppe with *Leymus* and *Carex*. Kastanozem soil. 50°28'14"N, 116°03'16"E. 29.07.2019.

[18] Near Pogibi, Sakhalin Oblast. *Larix* forest with *Pinus pumila*. Podzol soil. 52°11'47"N, 141°42'47"E. 11.07.2019.

[19] Pogibi, Sakhalin Oblast. Disturbed coniferous forest with *Pinus pumila* in suburban area. Podzol soil. 52°13'27"N, 141°39'19"E. 11.07.2019.

[20] Lazarev, Khabarovsky Krai. Typical taiga forest with *Larix gmelinii* predominance. Podzol soil. 52°11'18"N, 141°30'31"E. 12.07.2019.

[21] Near Lyublino, Primorsky Krai. Meadow/shrubland with *Carex*. Fluvisol soil. 44°54'15"N, 132°01'50"E. 07.08.2019.

[22] Lebedinoye, Primorsky Krai. Periodically flooded meadow with *Carex*. Fluvisol soil. 44°48'14"N, 132°45'23"E. 08.08.2019.

[23] 6 km north-east of Terney, Primorsky Krai. *Quercus*-*Betula* forest. Cambisol soil. 45°02'29"N, 136°40'56"E. 13.08.2019.

[24] Near Serebryanka river, Primorsky Krai. Sparse *Betula* forest. Cambisol soil. 45°06'42"N, 136°31'51"E. 14.08.2019.

[25] 10 km south of Terney, Primorsky Krai. *Quercus*-*Betula* forest. Cambisol soil. 44°57'19"N, 136°32'38"E. 15.08.2019.

[26] Gamova peninsula, Primorsky Krai. Sparse *Quercus* forest. Alfisol soil. 42°35'39"N, 131°11'44"E. 19.08.2019.

[27] Urup island, Sakhalin Oblast. Coniferous shrubland and meadow with *Sasa kurilensis*. Andosol soil. 45°53'32"N, 150°06'10"E. 09.08.2019.

[28] Eastern part of Staritskogo peninsula, Magadan Oblast. *Alnus* forest with high grass. Podzol soil. 59°30'08"N, 150°53'49"E. 28.07.2019.

[29] Suburban area near river Amur, Blagoveshchensk, Amur Oblast. Meadow/deciduous forest with *Prunus padus* and *Acer*. Cambisol soil. 50°17'18"N, 127°23'14"E. 05.08.2019.

[31] Near Kasatka Bay, Iturup island, Sakhalin Oblast. *Abies* forest and *Picea* forest with broadleaf undergrowth. Andosol soil. 45°00'10"N, 147°43'59"E. 20.08.2019.

## Results

We have found at least 12 genera and not fewer than 62 species in the RFE. No enchytraeids were found in sites [14] and [15], possibly due to the aridity of steppe habitats. The enchytraeid fauna consisted mostly of species from genera *Mesenchytraeus* Eisen, 1878 (15 species), *Henlea* Michaelsen, 1889 (10 species), *Fridericia*, and *Marionina* Michaelsen in Pfeffer, 1890 (nine species each), and *Bryodrilus* (seven species). *Cognettia* Nielsen & Christensen, 1959 is represented by four species, *Enchytraeus* Henle, 1837 by three species, *Achaeta* Vejdovský, 1878, *Euenchytraeus* Bretscher, 1906, and *Hemifridericia* Nielsen & Christensen, 1959 each by two species, and *Globulidrilus* Christensen & Dózsa-Farkas, 2012 and *Hemienchytraeus* Cernosvitov, 1934 each by a single species. The list of all the species, including those found in this study as well as those from the literature, with their localities is given in the Suppl. material 1: Table S1.

In addition to the species listed below, we also found several unidentified juvenile *Bryodrilus* at sites [27] and [28], *Fridericia* at [24, 26, 31], *Hemienchytraeus* at [29], *Hemifridericia* at [27], and *Mesenchytraeus* at [20, 23, 24, 26, 27, 28, 31]. For single records of species without a clear geographical reference, the approximate location is indicated directly in the list. Remarks on some species are also given below.

### A commented list of terrestrial Enchytraeidae of the Russian Far East

Genus *Achaeta* Vejdovský, 1878

1. Achaeta macroampullacea Dózsa-Farkas, Felföldi, Nagy & Hong, 2018 – 29, 31.

Specimens are fully consistent with the original description. Described from Jeju Island, Korea (Dózsa-Farkas et al. 2018), this is the first find of the species outside the original description.

1. Achaeta macrocyta Christensen & Dózsa-Farkas, 1999 – 8, 11, 12.

Genus *Bryodrilus* Ude, 1892

1. Bryodrilus arcticus (Bell, 1962) – 7, 8, 9, 10, 11, 12, 13.
2. Bryodrilus borealis Cejka, 1912 – 2, 13, 16.
3. Bryodrilus cejkai Nurminen, 1980 – 5.
4. Bryodrilus diverticulatus Cernosvitov, 1929 – 13, 23, 25, 26.

Enchytraeids from sites [23], [25], and [26] are not typical *B. diverticulatus*, but similar worms were already described from both Greenland and the Canadian Arctic Archipelago as a variety of *B. diverticulatus* with smaller male organs (Christensen and Dózsa-Farkas 2006).

1. Bryodrilus ehlersi Ude, 1892 – 7, 24.
2. Bryodrilus librus (Nielsen & Christensen, 1959) – 9, 10, 13.
3. Bryodrilus sp. A – 23.

**Figure 2.**
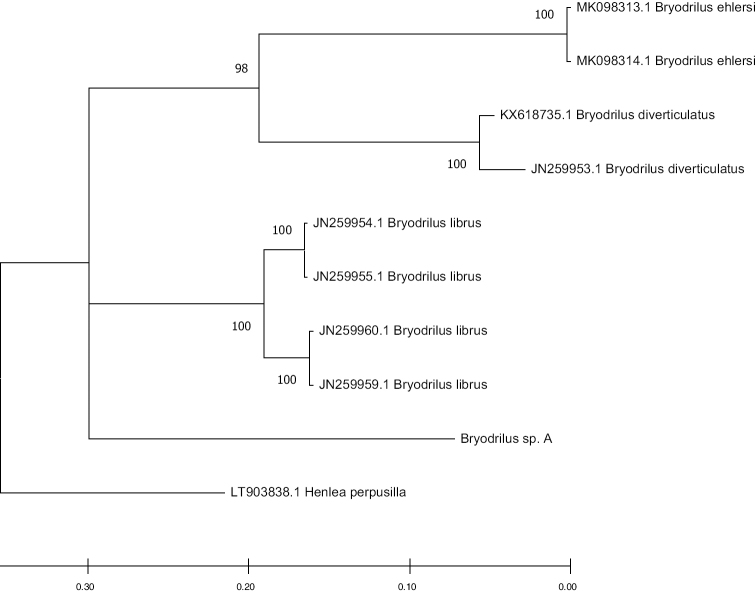
Phylogenetic tree of *Bryodrilus* species based on mtDNA COI obtained using Bayesian inference (BI). Numbers represent the posterior probabilities of the BI. Sequences obtained from NCBI GenBank are given with their accession numbers.

The resulting tree of BI phylogenetic analysis is shown in Figure 2. While the phylogenetic analysis gives support to *Bryodrilus* sp. A as a separate species of the genus, this inference may not be completely correct due to incomplete set of COI sequences available for the species of the genus. Taxonomic status of this species will be clarified in the near future.

Genus *Cognettia* Nielsen & Christensen, 1959

1. Cognettia glandulosa (Michaelsen, 1888) s.l. – 8, 11, 16.

This species was recently separated into two, *C. glandulosas.s.* and *C. varisetosa* (Martinsson, Erséus & Rota, 2015) (Martinsson et al. 2015b). The specimen from [16] morphologically corresponds to *C. glandulosas.s.*, but we cannot be sure of the specimens from the literature ([8, 11]).

1. Cognettia lapponica Nurminen, 1965 – 7, 8, 11, 12, 13, 18, 19, 20, 28.
2. Cognettia quadrosetosa Christensen & Dózsa-Farkas, 1999 – 13.
3. Cognettia sphagnetorum (Vejdovský, 1878) s.l. – 27, 31.

As this species is predominantly European and even in Europe it has large genetic variation (Martinsson et al. 2015a), we use the *sensu lato* approach. Found only in the Kuril Islands, it is most probably an introduced species.

Genus *Enchytraeus* Henle, 1837

1. Enchytraeus buchholzi Vejdovský, 1879 – 6, 7, 16, 23, 24, 26, 29, 31.
2. Enchytraeus christenseni Dózsa-Farkas, 1992 – 6.
3. Enchytraeus dichaetus Schmelz & Collado, 2010 – 16, 17, 21, 22, 24, 27.

Specimens are fully consistent with the original description. Some specimens at [16] have a short ectal duct. Some specimens at [21] have refractile vesicles in the coelomocytes. This is the first report of this species in the Asian part of Russia. Possibly, it is a Holarctic species with a range extending from North Africa (Rota and Healy 1994) to East Asia (Dózsa-Farkas et al. 2015) and Canada (Schmelz and Collado 2010).

Genus *Euenchytraeus* Bretscher, 1906

1. Euenchytraeus bisetosus Bretscher, 1906 – 11, 13.
2. Euenchytraeus piperi (Christensen & Dózsa-Farkas, 1999) – 8, 11, 12, 13, 23, 25, 28.

Genus *Fridericia* Michaelsen, 1889

1. Fridericia bisetosa (Levinsen, 1884) – 3, 24.
2. Fridericia bulboides Nielsen & Christensen, 1959 – 7, 8, 16, 21, 22, 24, 26, 27, 29.

**Figure 3.**
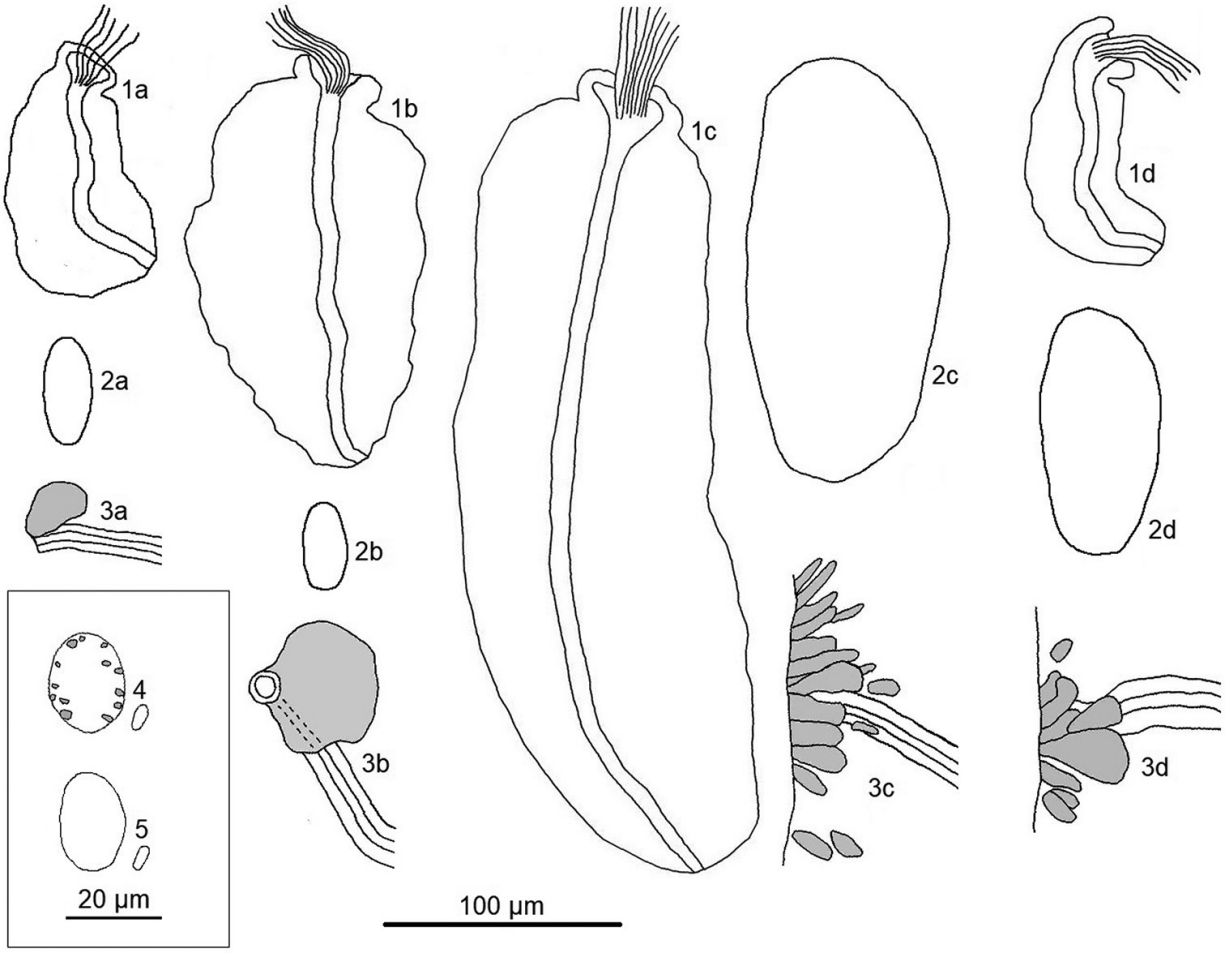
Morphological characteristics of four different types of *Fridericia bulboides*. For indexes **1a–3d** each numeral indicates a specific organ (**1** sperm funnel **2** male gland (only outline shown) **3** end of spermathecal ectal duct and spermathecal ectal gland), letter corresponds to morphological type (**a** “classical” *F. bulboides***b***F. bulboides* with large spermathecal ectal gland **c***F. bulboides* with big floppy sperm funnels, large male bulbs and compact glandular field near spermathecal ectal gland **d***F. bulboides* with medium-sized male bulbs and compact glandular field near spermathecal ectal gland) **4** coelomocytes of *F. bulboides* with refractile vesicles at coelomo-mucocyte periphery (types a, b) **5** coelomocytes of *F. bulboides* without refractile vesicles at coelomo-mucocyte periphery (types c, d).

**Figure 4.**
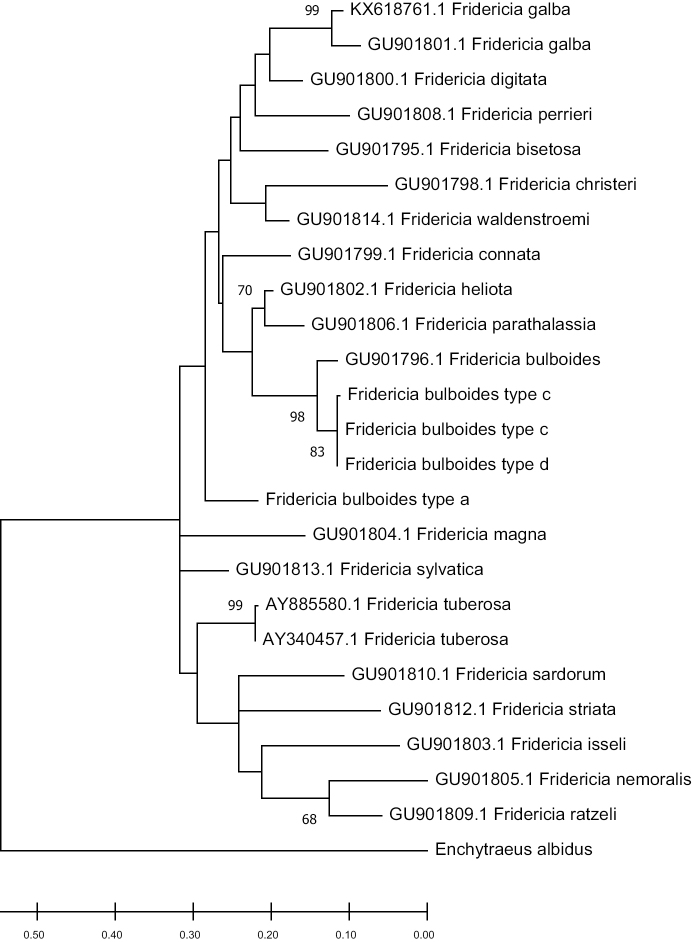
Phylogenetic tree of *Fridericia* species based on rRNA 16s obtained using Maximum likelihood (ML) method. Numbers represent bootstrap support values of ML analysis. Values lower than 65 are hidden. Sequences obtained from NCBI GenBank are given with their accession numbers.

Four morphological types were discovered in our material (Fig. 3): type a – typical *F. bulboides* ([7, 8, 16, 21, 22, 24 (partly), 26, 27]), type b – *F. bulboides* with large spermathecal ectal gland ([29]), type c – *F. bulboides* with big floppy sperm funnels, large male bulbs, and a compact glandular field near the spermathecal ectal gland ([24 (partly)]), and type d – *F. bulboides* with medium-sized male bulbs and a compact glandular field near the spermathecal ectal gland ([24 (partly)]). East-Asian *F. bulboides* with unusually large ectal gland are mentioned by Schmelz (2003). Those with the sperm funnel considerably larger and with the male copulatory organ and glandular structure at the orifice of the spermathecal ectal duct are found in Hungary (Cech and Dózsa-Farkas 2005). Our original phylogenetic analysis indicates that *F. bulboides* (types c, d) cannot be considered as separate species (Fig. 4). Although the final dendrogram has several events of unresolved phylogeny, there is relatively strong support for no division into separate species. On the other hand, *F. bulboides* (type a) from [24] is likely to be a new species of *Fridericia*, even with full morphological similarity with European specimens. However, such a claim demands further analysis with other molecular markers.

1. Fridericia callosa (Eisen, 1878) – 9, 10, 11, 13.
2. Fridericia cusanica Schmelz, 2003 – 27.

This species was described from Germany (Schmelz 2003) and is known from southern Europe (Schmelz and Collado 2010), but recently it was also recorded in Tibet (Lu et al. 2018). We found it from one of Kuril Islands. Thus, it is possibly a Holarctic species. This is the first report of *F. cusanica* in Russia.

1. Fridericia cf. cusanicaformis Dózsa-Farkas, Feldföldi & Hong, 2015 – 26, 29.

Differences from the original description (Dózsa-Farkas et al. 2015) are the absence of the spermathecal ectal gland and only one chaeta in ventral post-clitellar bundles. Our specimens were not fully mature, so these differences cannot be considered taxonomically significant. This is the first finding outside of Korea.

1. Fridericia cf. schmelzi Cech & Dózsa-Farkas, 2005 – 26.

The specimens from Primorsky Krai differed from the original description in the number of preclitellar nephridia (four). They had mostly three chaetae in lateral preclitellar bundles. The great distance to previously recorded localities (Hungary, Canada; Schmelz and Collado 2010) also raise doubts about the species' identity.

1. Fridericia sp. A – 29.

This is a new species. It will be described in an upcoming article.

1. Fridericia sp. B – 25.

This species does not fit the descriptions of the other species of *Fridericia* found in the RFE. This is a rare species, and more specimens needed for proper determination.

Genus *Globulidrilus* Christensen & Dózsa-Farkas, 2012

1. Globulidrilus riparius (Bretscher, 1899) s.l. – 16.

The specimens possess up to three chaetae in the ventral preclitellar bundles. This is the first find of this species and the genus in a terrestrial habitat in Russia. *Globulidrilus riparius* is possibly a trans-Palaearctic species having a range from Europe (Schmelz and Collado 2010) to Japan (Torii 2012). It was recently discovered than *G. riparius* is a complex of several species (Martinsson and Erséus 2018), but these are still undescribed.

Genus *Hemienchytraeus* Cernosvitov, 1934

1. Hemienchytraeus sp. A – 26.

This is the first find of the genus in Russia. The taxonomic status was not determined due to the poor condition of the extracted specimens.

Genus *Hemifridericia* Nielsen & Christensen, 1959

1. Hemifridericia parva Nielsen & Christensen, 1959 – 30.
2. Hemifridericia sp. A – 24.

This is probably a new species. More material is needed for a proper description, as it differs morphologically from the other three currently known *Hemifridericia* species (Christensen and Dózsa-Farkas 2006).

Genus *Henlea* Michaelsen, 1889

1. Henlea adiverticulata Christensen & Dózsa-Farkas, 1999 – 13.
2. Henlea conchifera Christensen & Dózsa-Farkas, 1999 – 10, 13.
3. Henlea diverticulata Cejka, 1912 – 2, 8, 10, 11.
4. Henlea glandulifera Nurminen, 1970 – 13, 27.
5. Henlea heleotropha Stephenson, 1922 – 13.
6. Henlea nasuta (Eisen, 1878) – 3, 5.
7. Henlea ochracea (Eisen, 1878) – 1, 5, 9.
8. Henlea perpusilla Friend, 1911 – 5, 7, 8, 10, 13, 14, 16, 22, 23, 25, 26, 29, 30, 31.

Specimens from [29] and [31] had only four pairs of fully developed preclitellar nephridia (6/7 – 9/10), whereas nephridia in 10/11 were rudimentary or (in some specimens from [31]) completely absent.

1. Henlea tolli Michaelsen, 1903 – 2, 4, 11, 13.
2. Henlea ventriculosa (d'Udekem, 1854) – 3, 5, 7, 8, 13, 16, 22, 23, 29.

Genus *Marionina* Michaelsen in Pfeffer, 1890

1. Marionina aporus (Stephenson, 1925) – 13.

This species has an uncertain status and was not mentioned by Schmelz and Collado (2012, 2015).

1. Marionina argentea (Michaelsen, 1889) s.l. – 9.

Christensen and Dózsa-Farkas (1999) wrote that the morphology of their specimens from the RFE is consistent with that accepted by Nielsen and Christensen (1959) before the separation of *M. argentea* into four species (Rota 2013). Thus, we use *sensu lato* approach here.

1. Marionina communis Nielsen & Christensen, 1959 – 30.
2. Marionina macrobulbi Christensen & Dózsa-Farkas, 1999 – 10.
3. Marionina minutissima Healy, 1975 – 12.
4. Marionina nordica Christensen & Dózsa-Farkas, 1999 – 13.
5. Marionina cf. nordica Christensen & Dózsa-Farkas, 1999 – 17.

Specimens are 5 mm long, always with 37 segments, and with spermathecal ectal glands absent; the chaetal formula is 3 – 2 : 3,2 – 2. The shape of the spermatheca generally fits the original description, but the chaetal pattern is unlike the original (2 – 2 : 3 – 3). This is most probably a new species.

1. Marionina southerni (Cernosvitov, 1937) – 31.

Genus *Mesenchytraeus* Eisen, 1878

1. Mesenchytraeus affinis Michaelsen, 1901 – 4.
2. Mesenchytraeus arcticus Bell, 1962 – vicinity of Kolyma (Christensen and Dózsa-Farkas 1999).
3. Mesenchytraeus asiaticus Eisen, 1904 – Chukotka (Eisen 1905).
4. Mesenchytraeus cejkai Cernosvitov, 1937 – 3.
5. Mesenchytraeus chaunus Piper, Maclean & Christensen, 1982 – 8, 12.
6. Mesenchytraeus diverticulatus Piper, Maclean & Christensen, 1982 – 7.
7. Mesenchytraeus gigachaetus Xie, 2012 – 23.

Our specimens generally fit the original description, differing only in the shape of the sperm funnels (collar wide, open, wider than sperm funnel body). This is the first find of *M. gigachaetus* outside China.

1. Mesenchytraeus kontrimavichusi Piper, Maclean & Christensen, 1982 – 8.
2. Mesenchytraeus konyamensis Michaelsen, 1916 – Chukotka (Michaelsen 1916).
3. Mesenchytraeus melanocephalus Christensen & Dózsa-Farkas, 1999 – 9, 12, 13.
4. Mesenchytraeus mirabilis Eisen, 1878 – 10.
5. Mesenchytraeus svetae Piper, Maclean & Christensen, 1982 – 8.
6. Mesenchytraeus torbeni Christensen & Dózsa-Farkas, 1999 – 9, 13.
7. Mesenchytraeus tundrus Piper, Maclean & Christensen, 1982 – 8.
8. Mesenchytraeus variabilis Cejka, 1914 – 3.

## Discussion

Representatives of *Mesenchytraeus* were found only in taiga forests and tundra. Thus, the biogeographic border between coniferous and deciduous forests, which runs across the RFE (Ryabinin et al. 2018), can also be the southern border of the unique Beringian fauna of enchytraeids, characterized by the dominance of large *Mesenchytraeus* (Piper et al. 1982). The RFE is apparently located at the contact area of three large enchytraeid faunas: (i) the Beringian fauna, (ii) the Euro-Siberian fauna, characterized by a great variety of *Fridericia* and *Henlea* spp. and the presence of small *Enchytraeus* spp., part of the *Enchytraeus buchholzi*-group (Nurminen 1973; Schmelz and Collado 2010), and (iii) the East-Asian fauna, characterized by a large number of endemic species (especially *Hemienchytraeus* spp.) (Dózsa-Farkas and Hong 2010) and possibly a few representatives of the boreal genus *Cognettia* (Zhang et al. 2018). Thus, biogeographically, enchytraeids of the RFE should be studied not as single fauna of the whole region, but as a combination of several, partially overlapping faunas.

A list of enchytraeids of the RFE is prepared for the first time and consists of at least 62 species. This number is about half the number of species in Germany (Römbke et al. 2013), mainly because of the lack of knowledge of the RFE. Thus, this article is just a first step, with some unaddressed questions on the enchytraeid fauna for future studies. Some species, especially those that were described in the 1900–1910s, need taxonomic revision because their descriptions are very poor and inconsistent with modern science. Based on our molecular results, we suggest two potentially new species. Undeniably, the Russian Far East, especially its southeastern part, is of great interest as a possible location for new species of enchytraeids.

## Supplementary materials

Supplementary material 1 Table S1 Data type species data https://binary.pensoft.net/file/439401This dataset is made available under the Open Database License (http://opendatacommons.org/licenses/odbl/1.0/). The Open Database License (ODbL) is a license agreement intended to allow users to freely share, modify, and use this Dataset while maintaining this same freedom for others, provided that the original source and author(s) are credited.Maksim I. Degtyarev, Iurii M. Lebedev, Ksenia G. Kuznetsova, Konstantin B. Gongalsky
